# Galectin-9 and Tim-3 in gastric cancer: a checkpoint axis driving T cell exhaustion and Treg-mediated immunosuppression independently of anti-PD-1 blockade

**DOI:** 10.3389/fimmu.2025.1600792

**Published:** 2025-07-01

**Authors:** Charlotte N. Hill, Gabriela Maita, Camille Cabrolier, Constanza Aros, Ana Maria Vega-Letter, Pamela Gonzalez, Alexis M. Kalergis, Patricia Luz-Crawford, Gareth I. Owen

**Affiliations:** ^1^ Facultad de Ciencias Biológicas, Pontificia Universidad Católica de Chile, Santiago de Chile, Chile; ^2^ Millenium Institute for Immunology and Immunotherapy, Santiago de Chile, Chile; ^3^ Laboratorio de Inmunología, Centro de Investigación e Innovación Biomédica, Facultad de Medicina, Universidad de los Andes, Santiago de Chile, Chile; ^4^ Center of Interventional Medicine for Precision and Advanced Cellular Therapy (IMPACT), Santiago, Chile; ^5^ Escuela de Ingeniería Bioquímica, Pontificia Universidad Católica de Valparaíso, Valparaíso, Chile

**Keywords:** gastric cancer, immune checkpoint blockade, galectin-9, TIM-3, anti-PD1

## Abstract

Immune checkpoint inhibitors have significantly advanced the treatment of gastric cancer (GC), yet therapeutic resistance remains common due to the immunosuppressive tumor microenvironment and redundancy among inhibitory checkpoints. Tim-3 (HAVCR2) is an emerging immune checkpoint receptor implicated in tumor immune evasion. However, the role of its ligand, Galectin-9 (Gal-9, LGALS9), in GC pathogenesis and therapy resistance remains poorly understood. We performed bioinformatic analysis of The Cancer Genome Atlas (TCGA) stomach adenocarcinoma (STAD) dataset to assess LGALS9 and HAVCR2 expression and their clinical correlations. We also evaluated associations between LGALS9 expression and immune cell signatures. Functional ex vivo assays were conducted to investigate the effects of Gal-9 on CD8⁺ T cell function and Treg suppressive activity in the context of Tim-3 signaling. Our analysis revealed that both LGALS9 and HAVCR2 are upregulated in gastric tumors and associated with poor patient survival. HAVCR2 expression was significantly higher in invasive adenocarcinomas. LGALS9 expression strongly correlated with signatures of CD8⁺ T cell dysfunction and increased infiltration of regulatory T cells (Tregs). Functionally, Gal-9 promoted Treg suppressive activity and CD8⁺ T cell dysfunction ex vivo through Tim-3 engagement, independently of PD-1 signaling. These findings suggest that Gal-9 contributes to immune evasion in GC by promoting Treg expansion and CD8⁺ T cell exhaustion, potentially driving resistance to anti-PD-1 therapy. We propose circulating Gal-9 as a candidate biomarker of anti-PD-1 resistance and support the rationale for combined blockade of PD-1 and Tim-3 to enhance immunotherapeutic efficacy in GC.

## 1 Background

Gastric cancer (GC) takes the fifth place on most common cancers worldwide and it is currently the third cause of death by malignant tumors (1–3). In Chile, GC is a principal cause of cancer death (2, 3), with most patients being diagnosed with advanced or metastatic disease (4, 5). While the five-year survival rate for GC patients is 30.4%, this rate falls to approximately 5% in metastatic GC patients, reducing life expectancy to less than a year (6). In the absence of a standard-of-care treatment for advanced or recurrent GC after chemotherapy failure, there is a clinical requirement for new treatment options.

Pembrolizumab, a PD-1 blocking antibody, was granted accelerated approval for the treatment of patients with recurrent locally advanced or metastatic gastric or gastroesophageal junction adenocarcinoma (7). Although this therapy showed a remarkable 56% overall response rate (ORR) on the KEYNOTE-052 trial, the most recent published study shows a 22.7% ORR (8). Currently the only companion diagnostic available is an FDA approved test to determine tumor PDL-1 expression, but despite this selection 80% of patients show no clinical benefit (8), losing their opportunity to receive an effective treatment and suffering the elevated economic burden of this treatment. Similar results have been shown on CHECKMATE trials for another αPD-1 antibody, Nivolumab. In a phase 1/2 study in chemotherapy-refractory GC patients, second line Nivolumab treatment delivered a 26% ORR (44% ORR in PD-L1^+^ tumors) (7). Thus, while checkpoint therapy is offering enhanced overall survival to a significant number of patients, a better knowledge of GC immune escape mechanisms is required to identify possible predictive biomarkers and design new therapeutic approaches to overcome resistance. Of note, most proposed biomarkers are based on static measures and therefore cannot be evaluated through time and hence fail to rule out acquiring resistance.

Tim-3 is expressed on the most dysfunctional subset among tumor-infiltrating CD8^+^PD1^+^ T cells in cancer (9–11), and its upregulation in T cells exacerbates tumor progression in murine models (12). Therefore, Tim-3 has been proposed as a marker that identifies both terminally differentiated effector cell and irreversibly exhausted T cells (13–17). Strikingly, Tim-3 upregulation can be observed after αPD1 therapy (18). Additionally, the simultaneous blockade of Tim-3 and PD1 increases T cell responses compared with anti-PD 1 monotherapy (9, 11, 19–22). On the other hand, Tim-3^+^Treg are enriched in the tumor and correlate with tumor aggressiveness and progression (23–25). Tim-3^+^T_reg_ display an enhanced immune-suppressive function that can be abrogated by Tim-3 blockade (23, 26–28). In fact, αTim-3 synergizes with αPD1 therapy to reduce T_reg_ infiltration and increase CD8^+^ T cell infiltration (9). Tumor Tim-3^+^T_reg_ accumulation precedes CD8^+^ T cell dysfunction, and that their early depletion prevents CD8^+^ T exhaustion (23).

Gal-9 expression is found in 57% of gastric tumors, a key Tim-3 ligand associated with poor prognosis (29–31). Moreover, Tim-3 has also been proposed as a prognostic marker for solid tumors including GC, with high levels of Tim-3 expression associated with poor survival (32). However, the presence of an immune-suppressive tumor microenvironment and redundancy between inhibitory immune checkpoints may be responsible for why a high percentage of GC patients do not show clinical benefit of ICB.

An emerging target is the inhibitory receptor Tim-3 which has been reported to serve as Galectin-9 receptor. Galectin-9 has been widely implicated in CD8+ T cell exhaustion; however, its role in gastric cancer has not been previously characterized. Our findings demonstrate that Gal-9 is sufficient to enhance immune checkpoint expression even in the presence of anti-PD-1 blockade *ex vivo*, suggesting that it may contribute to therapy resistance. Further clinical studies are required to validate its role and assess its potential as a predictive biomarker in a clinical setting.

## 2 Methods

### 2.1 TCGA database bioinformatics analysis

The bioinformatics analysis of gastric cancer data of patients with gastric adenocarcinoma from TCGA Database was performed with TIMER (Tumor Immune Estimation Resource; ^©^ X Shirley Liu Lab & Jun Liu Lab 2018). Specifically, gene expression quartiles were used to define high and low expression, and z-score > 2 was used as a threshold for overexpression relative to normal tissue. Spearman and Pearson’s regression analysis were performed considering a p-value of 0.05 or less significant. TIMER analysis considered data of 387 tumor samples from 1 data set (Stomach Adenocarcinoma 415 dataset) of untreated patients from the TCGA database. Tumor infiltrating-T_reg_ and CD8^+^ exhaustion signatures were calculated as previously described (33, 34). Briefly, each signature was calculated using the mean z-scores of gene expression values corresponding to each gene least. Then, specific correlation of LGALS9 levels (TPM) with molecular signatures were performed by spearman’s regression analysis.

Overall Survival (OS) and Progression-Free Survival (PFS) and Post Progression Survival (PPS) from patients was obtained from the TCGA STAD 2018 data set along with HAVCR2 and LGALS9 mRNA levels. High and low levels were defined as the upper and low quartile respectively. Hazard ratio (HR) was calculated using LogRank (Mantel-Cox) and Gehan-Breslow-Wilcoxon tests on GraphPad^®^Prism 9.0.

### 2.2 Gastric cancer and epithelial cell lines culture

The gastric cancer cell line AGS and the gastric epithelial cell line GES-1 were cultured in 10% FBS supplemented RPMI 1640 and transfected using a CMV3-LGALS9 expression vector or empty vector as control. Transfections were performed during 4–6 hours in OPTIMEM media using a transfection solution with 1:1 DNA: Fugene. Gal-9 expression was assessed by western blot (human anti-Gal9, CST) and ELISA (Gal-9 DuoSet R&D). Besides transfections, recombinant human Galectin-9 (rhGal-9, R&D) was used to treat cells. After transfection or rhGal-9 treatment, viability was evaluated by WST-1 assay (Sigma), migration and invasion assay were performed as previously described (35) in the presence or absence of 30mM α-Lactose (Sigma).

### 2.3 Primary T cell cultures and Flow cytometry

T cells were isolated from peripheral blood mononuclear cells (PBMC) from healthy donors with written consent and ethical approval from the Ethical Committee of the Pontificia Universidad Católica de Chile. First, PBMCs were isolated by Ficoll gradient (Ficoll-Plus^®^), then T cells were enriched by magnetic separation using the Untouched Human Pan T Cells isolation kit^®^ (Life Technologies) according to manufacturer’s indications. T cells were continuously stimulated with Dynabeads^®^ CD3/CD28, 10ng/mL IL-2 and rhGal-9 (0.25, 0.5, and 1μg/mL) in presence or absence of blocking antibodies: 20μg/mL αTim-3 (Biolegend) and 20μg/mL αPD-1 (InVivoMab^®^) and cultured for 5 days in T cell media (10ng/mL IL-2 (Biolegend), 10% FBS, 1% Penicllin-Streptomycin, 1% Glutamine, 50μM B-Mercaptoethanol, 50nM HEPES supplemented RPMI Glutamax). After culture cells were stained with LIVE/DEAD™ Fixable Near-IR Dead Cell Stain Kit^®^ (LifeTechnologies) and labelled antibodies against CD4, CD8, CD127, CD25, PD-1, Tim-3, Lag-3, IFNγ, and Foxp3. For IFNγ staining, cells were stimulated with phorbol 12-myristate 13-acetate (PMA, Sigma), Ionomycin (Sigma) and Brefeldin A (Biolegend) for 4 hours before staining. Results were obtained by flow cytometry using FACS BD –Canto II and FACS DIVA and results were analyzed using FlowJo 10.5.

### 2.4 ELISA assay

Galectin-9 ELISA assay was performed using Gal-9 DuoSet kit according to manufacturer instructions. Briefly, MaxiSorp plates (Falcon) were coated overnight with anti-Gal-9 in the PBS-BSA solution at RT. Then washed with PBS-Tween, blocked for 1h at and incubated with cell conditioned media for 2h at RT, after incubation plates were washed and incubated with capture antibodies and streptavidin. Colorimetric assay using TMB was performed and after 15 min absorbance was measured at 450nm and 690nm and the concentration of samples was calculated by applying a 4-PL regression to the standard curve.

### 2.5 Statistical analysis

Statistical analyses were performed using GraphPad Prism 9. For comparisons between two independent groups, the non-parametric Mann–Whitney U test was applied due to small sample sizes and non-normal distribution. When comparing more than two groups, Kruskal–Wallis tests followed by Dunn’s *post hoc* test were applied to account for non-normal distributions and multiple comparisons. Correlations in gene expression data from the TCGA-STAD cohort were assessed using Spearman’s rank correlation. Kaplan–Meier survival analyses were conducted using the log-rank (Mantel–Cox) test, with hazard ratios calculated for high vs. low expression quartiles. A p-value < 0.05 was considered statistically significant.

## 3 Results

### 3.1 Elevated levels of LGALS9 and HAVCR2 are associated with cancer progression and reduced patient survival

**Figure 1:**
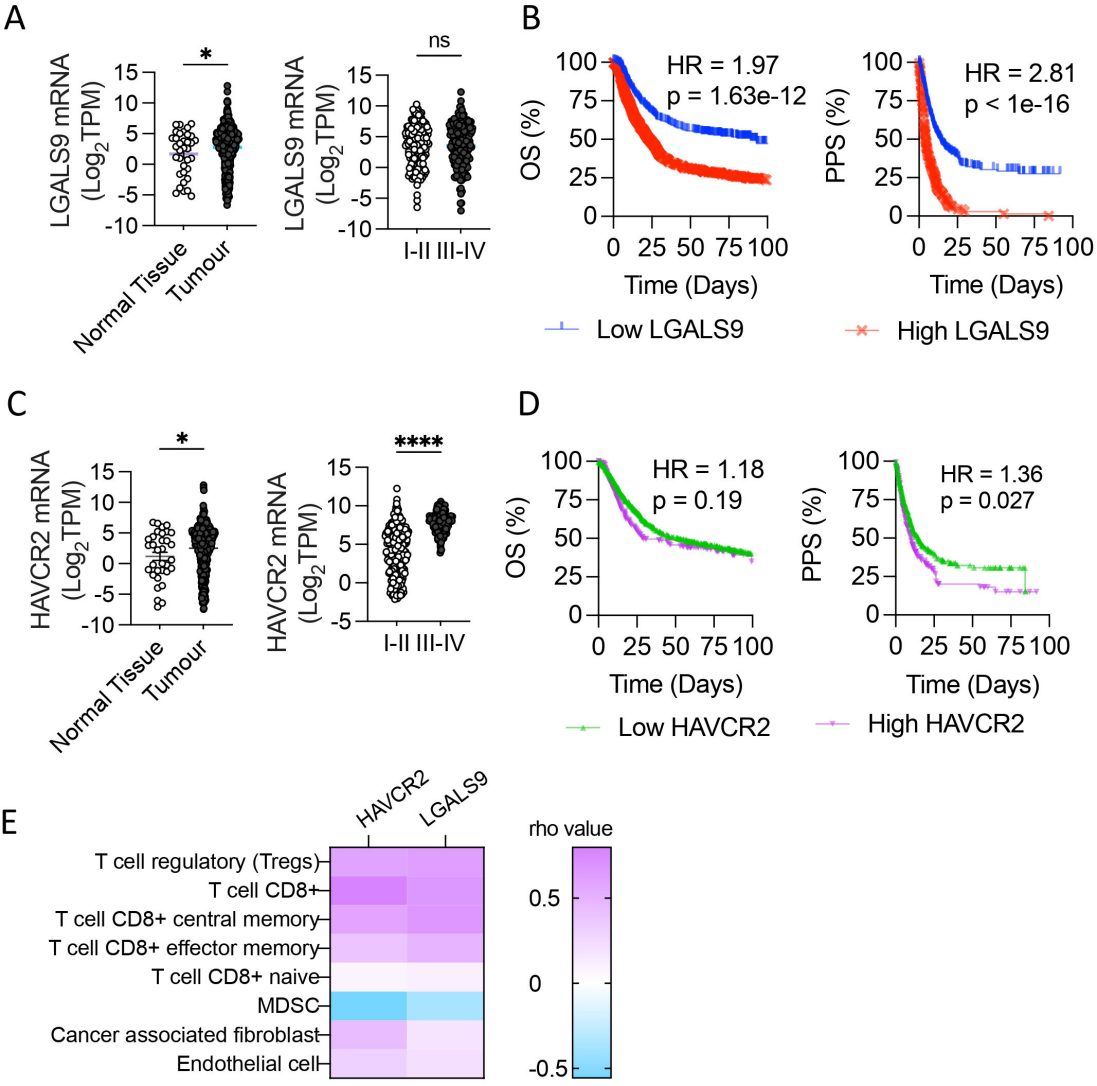
Galectin-9 and Tim-3 levels in Stomach Adenocarcinoma associate with patient 583 outcome. Galectin-9 (LGALS9) and Tim-3 (HAVCR2) mRNA levels were evaluated in normal gastric 584 tissue samples and tumors from the TCGA database (STAD 2018). LGALS9 and HAVCR2 levels in 585 normal tissue and tumor and its levels among stages **(A, C)**, *p,0.05, ****p<0.0001. *unpaired t-Student test*. 586 Overall Survival (OS) and Post Progression Survival (PPS) depending on LGALS9 **(B)** and HAVCR2 587 **(D)** levels. Pearson correlation of HAVCR2 and LGALS9 mRNA levels with tumor 588 microenvironment cell gene signature (TIMER®) **(E)**. Gehan-Breslow-Wilcoxon test and LogRank test. 589 HR, Hazard Ratio; TPM, Transcripts Per Million.

Galectin-9 (LGALS9) and Tim-3 (HAVCR2) mRNA levels were evaluated in registered normal gastric tissue samples (n=35) and tumors (n=379) from the TCGA database (STAD 2018). LGALS9 was significantly increased in tumor samples (2.773 ± 0.18) compared to normal tissue (1.708 ± 0.61), while it did not show significant differences between stages; stage I and II displayed a mean level of 6.412 ± 0.17 and stages III and IV a mean of 7.723 ± 0.08 (**Figure 1A**). HAVCR2 levels were also significantly higher on tumor samples (2.517 ± 0.19) than in normal tissue (1.188± 0.69) (**Figure 1D**). Unlike its ligand, HAVCR2 levels were increased in advanced-stage (7.723 ± 0.08) compared to early-stage disease (6.419 ± 0.08) (**Figure 1C**).

Given that LGALS9 and HAVCR2 levels were upregulated in STAD and that HAVCR2 levels were associated with advanced stages, we evaluated if higher levels of mRNA expression were associated with poor clinical outcomes (**Figures 1B, D**). The Overall Survival (OS) was shorter in patients with the highest LGALS9 quartile, with a median survival of 89.43 compared to 23.5 months in the lowest quartile (p<0.0001), with a Hazard Ratio (HR) of 1.97 (**Figure 1B**). In a similar pattern, the Post Progression Survival (PPS) of the highest and lowest LGALS9 quartile presented a median survival of 4.3 and 13.8 months, respectively, with an HR of 2.81 and p<0.0001. OS of HAVCR2 highest and lowest quartile levels had a median survival of 29.2 and 51.8 months respectively, with a HR=1.189 and p=0.186 (**Figure 1D**). On the other hand, PPS of the HAVCR2 highest quartile presented a median survival of 10.3 months, significantly shorter that the lowest quartile of 14.8 months (HR=1.36, p=0.027).

As LGALS9 and HAVCR2 genes are associated with immune regulation, we evaluated the correlation of these transcripts’ abundance with the gene signature of tumor microenvironment cells Cibersort ABS on TIMER^®^. Both genes strongly associate with tumor T cells, including CD8^+^ and Treg, obtaining rho values > 0.5 for these gene signatures (**Figure 1E**).

### 3.2 Gal-9 promotes cell migration and invasion *in vitro*

**Figure 2:**
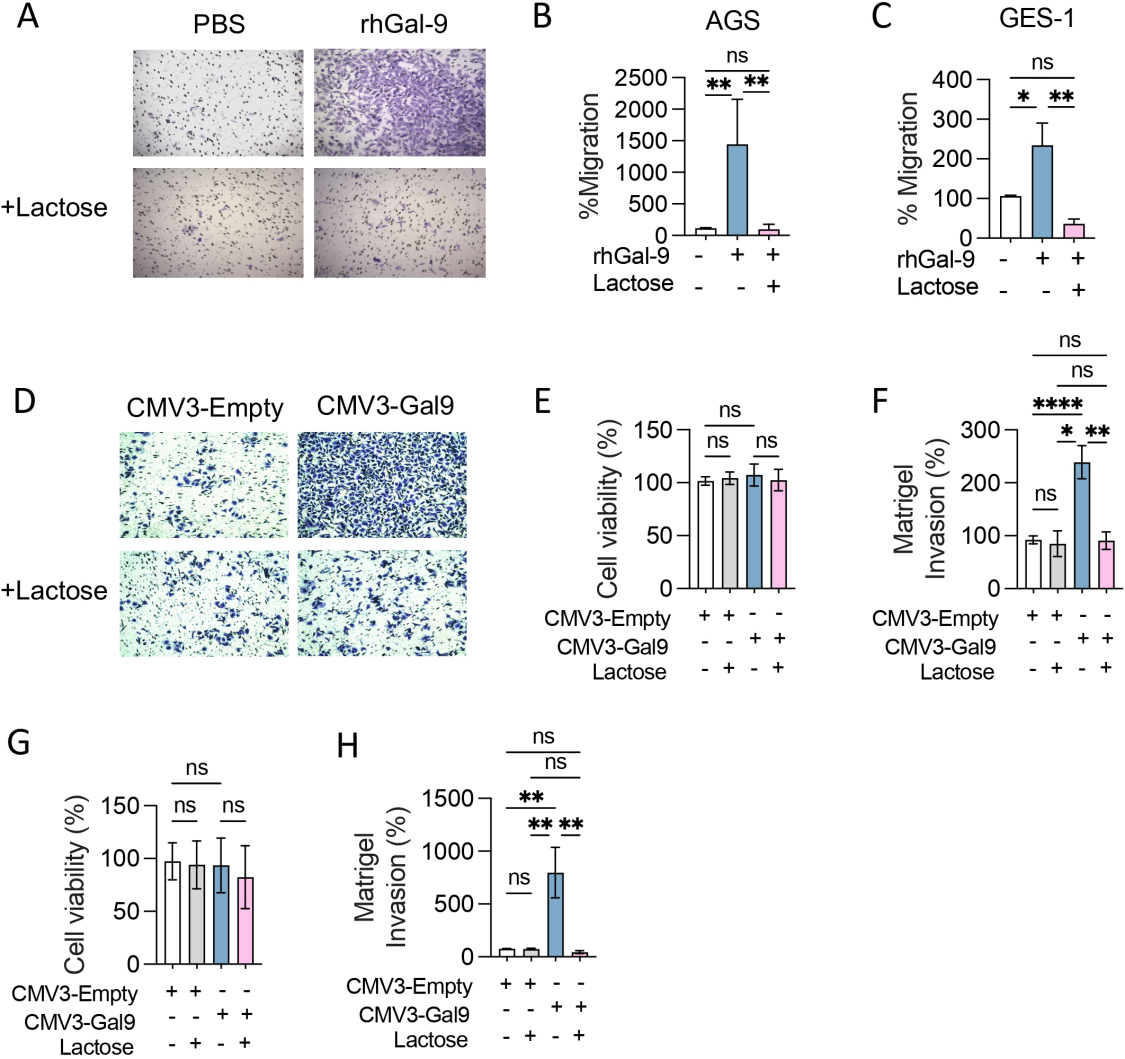
Galectin-9 promotes cancer and epithelial cell migration and invasion *in vitro*. Transwell migration **(A-C)** and Matrigel invasion assays **(D-H)** were performed with AGS and GES-1 cell lines in presence of recombinant human Galectin-9 (rhGal-9) or upon Galectin-9 overexpression (CMV3-Gal9). Lactose 30mM was used to block Gal-9 glycan binding regions. Representative photographs of stained membranes are shown **(A, D)**. Cell viability after transfection was measured by MTS assay in AGS **(E)** and GES-1 **(G)** cell lines. Matrigel invasion of transfected AGS **(F)** and GES-1 **(H)** was quantified. Migration and Invasion percentages were calculated considering PBS or CMV3-Empty as control. Results presented as mean and SD, *p<0.05, ** p<0.01, ****p<0.0001 Kruskal-Wallis, U-Man Whitney, n=3.

To determine the consequences of Gal-9 overexpression in GC cells, we transfected AGS cells with a Gal-9 expression vector (CMV3-Gal9) or empty vector as control (CMV3-Empty). CMV3-Gal9 transfection led to a 10-fold increase in protein expression and secretion without altering cell viability or proliferation (**Supplementary Figure S1**). We evaluated if Gal-9 overexpression could enhance cancer cell migration and invasion. The addition of rhGal-9 to the AGS cell line caused a significant increase in cell migration (**Figures 2A, B**), which was also observed in the gastric epithelial cell line, GES-1 (**Figure 2C**). Interestingly, when cells were treated with a-Lactose to block extracellular Gal-9 sugar-binding domains, the increase in cell migration was prevented in both cell lines (**Figures 2E, G, H**). Similarly, Gal-9 overexpressing AGS and GES-1 cells displayed a significant increase in invasion, which was effectively prevented by α-Lactose (**Figures 2D, F**), suggesting that Gal-9 promotion of cell migration and invasion may be both physiological and pathophysiological.

### 3.3 Galectin-9 associates with tumor T_reg_ infiltration and promotes Tim-3^+^ Treg expansion and suppressive capacity *in vitro*

**Figure 3:**
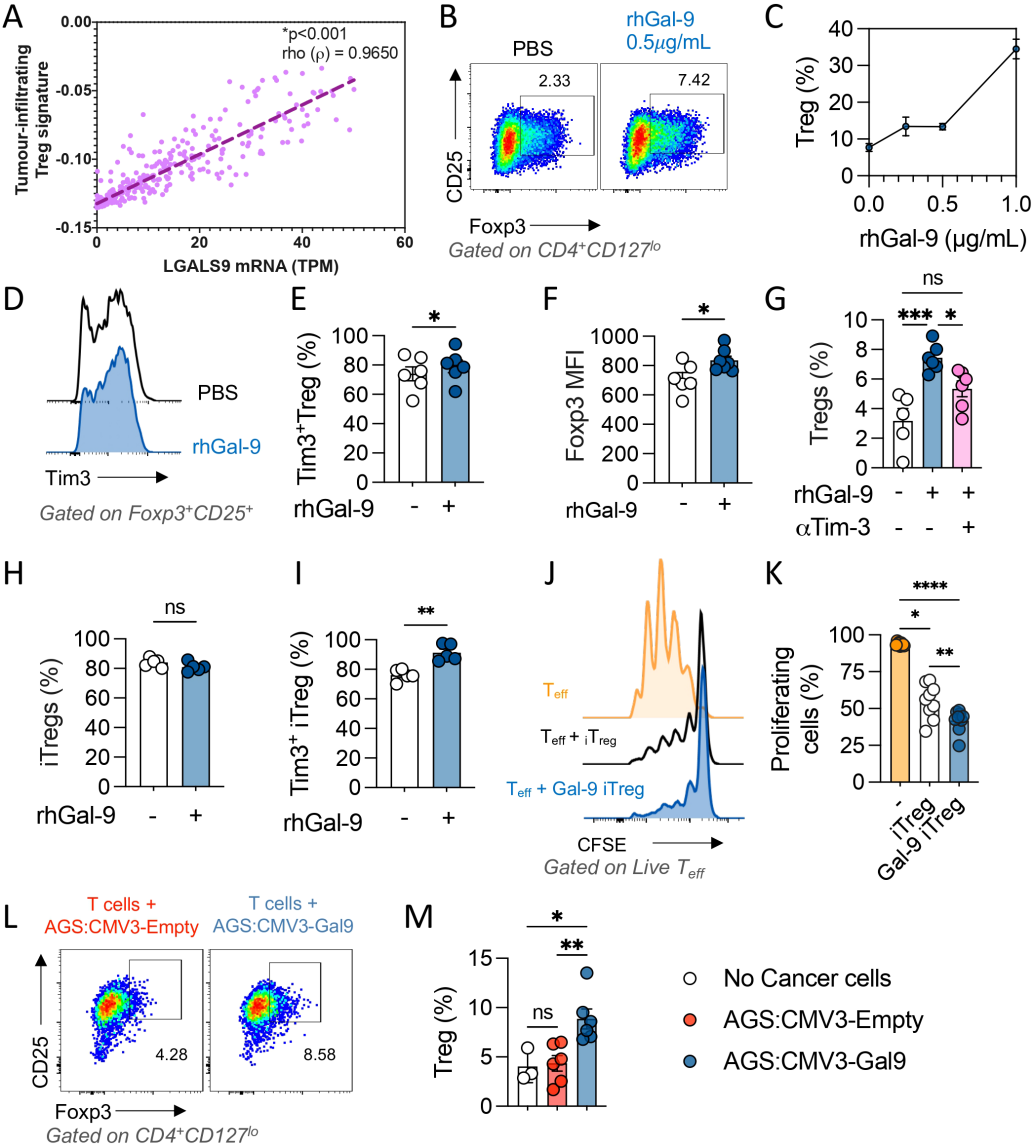
Galectin-9 associates with tumor Treg infiltration and promotes Tim-3^+^ iTreg expansion and suppressive capacity *in vitro*. Tumor infiltrating Treg transcriptional signature was determined using the STAD 2018 TCGA dataset and its correlation with LGALS9 mRNA levels was evaluated through Spearman’s regression, Rho value=0.3642 and p<0.001 **(A)**. CD3+ T cells were enriched and activated with αCD3/αCD28 in the presence of rGal-9, a dose dependent increase in Treg frequency was observed **(B, C)**. rhGal-9 upregulated Tim-3 **(D, E)** and Foxp3 **(F)** in Treg cells. The addition of an αTim-3 blocking antibody on Treg frequency was also assessed **(G)**. Naïve CD4 T cells were isolated and iTreg were generated *in vitro* in presence of rhGal-9 **(H)**, frequency of Tim3^+^ iTreg **(I)** was assessed. The suppressive capacity of then iTreg was then evaluated in a suppression assay by coculture with CTV stained Tconv **(J, K)**. Coculture of CD3^+^ T cells with Gal-9 expressing cancer cells (AGS CMV3-Gal9) or control cancer cells (AGS CMV3-Empty) **(L, M)**. Results presented as mean and SE *p<0.05; **p<0.01; ***p<0.001; ****p<0.0001 Mann Whitney test or Kruskal Wallis – Mann Whitney.

As Cibersort utilizes molecular signatures based on natural T_reg_ and mice models, we performed a gene set enrichment analysis based on a tumor infiltrating-T_reg_ signature (34). We found a strong association between LGALS9 levels and this T_reg_ signature, with a Rho value of 0.965 and p<0.001 after Spearman’s regression (**Figure 3A**). As LGALS9 levels strongly correlated with tumoral-T_reg_’s signature, we treated T cells with increasing concentrations of rhGal-9 (0.25, 0.5 and 1.0µg/mL) and observed a dose-dependent increase of CD4^+^CD127^lo^CD25^+^Foxp3^+^ T_reg_ frequency (**Figures 3B, C**). Noteworthy, rhGal-9 addition did not impact CD3+ T cell viability (**Supplementary Figure S2**). Therefore, Gal-9 may promote T_reg_ expansion through Tim-3 signaling. In fact, we found that Tim3^+^T_reg_ is increased and that Foxp3 is upregulated upon Gal-9 engagement (**Figures 3D–F**). To determine if this effect was dependent on Tim-3 engagement, we used a blocking antibody targeting Tim-3, which prevented the rhGal-9 mediated increase in T_reg_ frequency among total T cells (**Figure 3G**). To determine if Gal-9 could promote Treg induction as previously described in murine models, we treated CD4+ naive cells with rhGal9 (**Supplementary Figure S3**). Interestingly, in this setting Gal-9 failed to increase T_reg_ induction from naïve CD4+ T cells, however, it enriched the Tim3^+^iTreg population and enhanced the suppressive capacity of the induced Tregs (**Figures 3H–K**). Additionally, only Gal-9 overexpressing cancer cells expanded the Treg population (**Figures 3L, M**).

**Figure 4:**
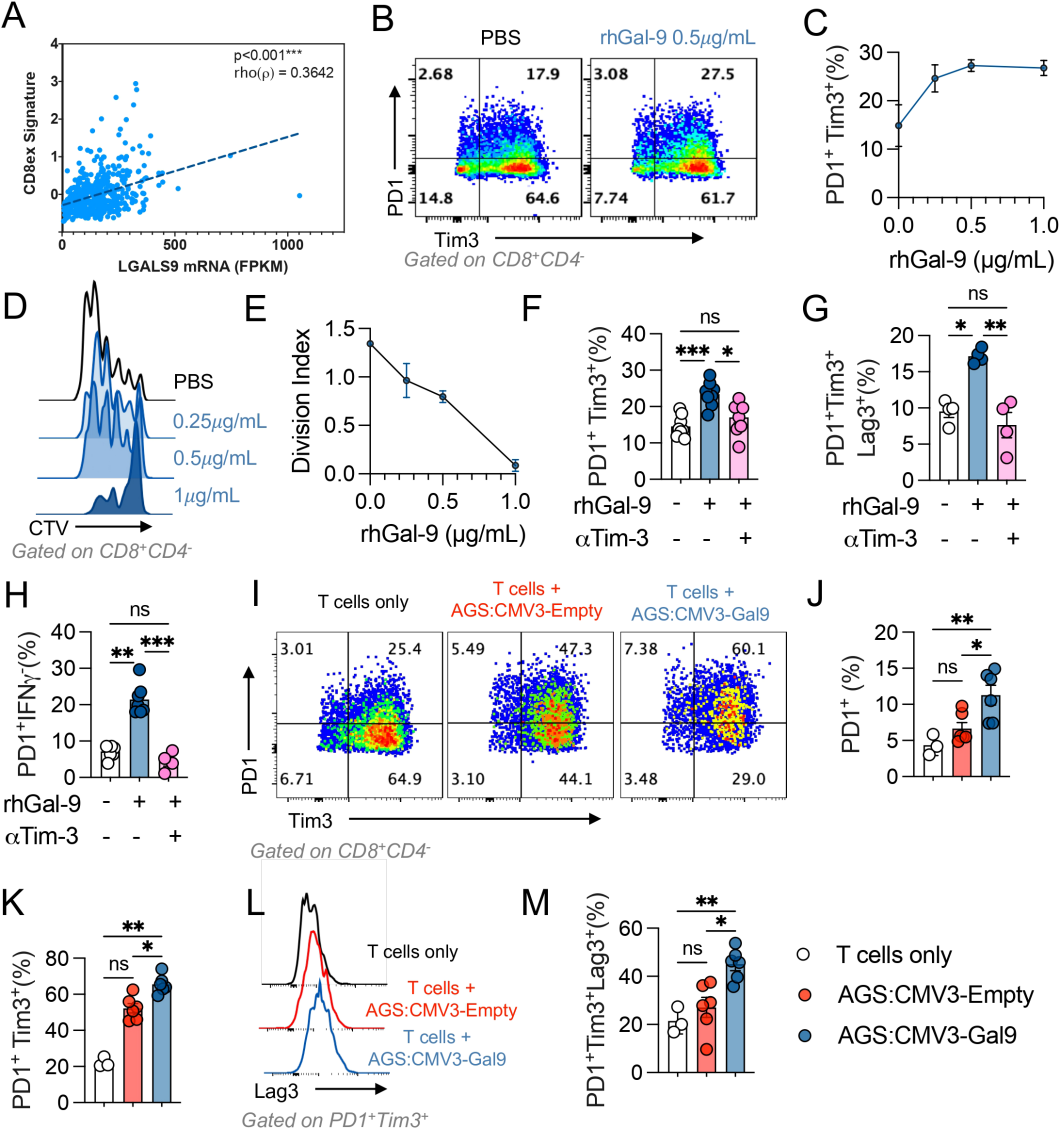
Galectin-9 associates with CD8+ T cell exhaustion in stomach adenocarcinoma. Correlation between LGALS9 levels and an exhausted CD8+ (CD8ex) signature on the STAD 2018 TCGA dataset was performed, Spearman’s regression, Rho value=0.3642 and p<0.001 **(A)**. Isolated CD3+ T cells from healthy donors were activated with αCD3/αCD28 beads and treated with rhGal-9 or vehicle (PBS) **(B, C)** in the presence or absence of an aTim3 blocking antibody. The frequency of PD1+Tim3+ **(B, C)** and the division index **(D, E)** was assessed by flow cytometry. To determine the effect of the addition of aTim- 3, the frequencies of PD1+Tim3+ **(F)**, PD1+Tim3+LAG3+ **(G)** and PD1+IFNg+ **(H)** CD8+T cells was assessed (n=8). Gal-9 expressing (CMV3-Gal9) or control s (CMV3-Empty) AGS cells were cocultured with aCD3/aCD28 activated CD3+ T cells, after 72h the frequencies of PD1+, PD1+Tim3+ **(I-K)**, PD1+Tim3+LAG3+ **(L, M)** CD8+T cells were assessed (n=5). Results presented as mean and SE *p<0.05; **p<0.01; ***p<0.001 Kruskal Wallis – Mann Whitney.

Gal-9 associates with CD8+ T cell exhaustion in Stomach Adenocarcinoma and promotes an exhausted like phenotype in blood derived CD8+ T cells. Given that the Gal-9 mRNA levels are associated with a CD8^+^ 220 T cell dysfunction signature in gastric tumours (**Figure 4A**), we evaluated if Gal-9 promoted the frequency of dysfunctional CD8^+^ T cells *in vitro*. T cells were isolated with informed consent from healthy donors (n=8) and stimulated with αCD3/αCD28 activating beads in presence of rhGal-9 at increasing concentrations (0.25, 0.5 and 1µg/mL). Although Gal-9 effectively decreased total CD8^+^ T cell frequency (data not shown), this population was significantly enriched in PD-1^+^Tim-3^+^ exhausted-like T cells and dose-dependently decreased cell division (**Figures 4B–D**). We assessed if a Tim-3 blocking could prevent CD8^+^ T cells exhaustion by measuring the frequency of PD-1^+^Tim-3^+^, PD-1^+^Tim-3^+^Lag-3^+^, and PD-1^+^IFNy^-^ subpopulations. Gal-9 significantly increased these populations of CD8^+^ T cells, which was completely abolished by the blockade of Tim-3 (**Figures 4E, F**). Similar results were observed when isolated CD8^+^ T cells were stimulated with Gal-9 (**Supplementary Figure S2**).

We then evaluated if Gal-9 expressing cancer cells were capable of increasing CD8^+^ T cell exhaustion. When co-culturing Gal-9 overexpressing cancer cells with T cells, we observed a significant increase in the percentage of CD8^+^PD1^+^, CD8^+^PD1^+^Tim3^+^, and CD8^+^PD-1^+^Tim-3^+^Lag-3^+^ T cell populations (**Figures 4G–L**). These results indicate that Gal-9 expressing cancer cells can promote CD8^+^ T cell exhaustion and T_reg_ expansion.

### 3.4 The Galectin-9 mediated increase in exhausted-like CD8^+^ T cells is independent of PD-1

**Figure 5:**
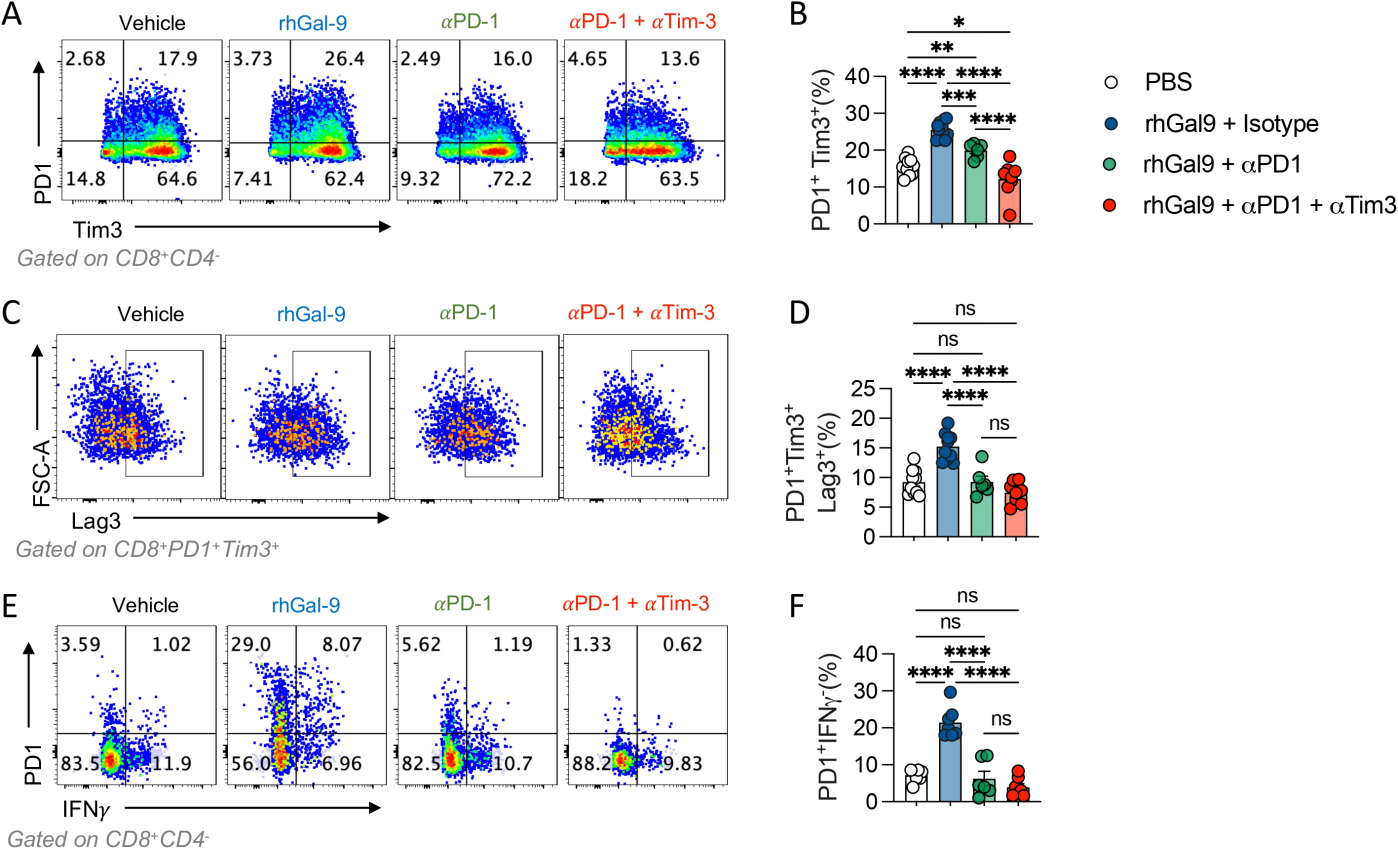
Galectin-9 increases PD1^+^Tim3^+^ T cells in the presence of αPD1 blocking antibody. T cells isolated from healthy donors were activated with αCD3/αCD28 beads and treated with rhGal9 in presence or absence of αPD1 blocking antibodies or a combination of αPD1 and αTim3 antibodies. After 96h, cells were harvested and stained for flow cytometry. PD1^+^Tim3^+^ **(A, B)**, PD1^+^Tim3^+^Lag3^+^ **(C, D)** and PD1^+^IFNγ^+^ **(E, F)** frequencies were assessed. Results presented as mean and SE *p<0.05, **p<0.01, ***p<0.001, ****p<0.0001 n=5, Kruskal Wallis, Mann Whitney.

Given that Gal-9 increased exhausted-like CD8^+^ T cells populations, we evaluated if this could be maintained upon αPD-1 blockade. The percentage of CD8^+^ T cells and the exhausted-like populations PD1^+^Tim3^+^, PD1^+^Tim3^+^LAG3^+^, PD1^+^IFNy^-^ were assessed by flow cytometry (**Figure 5**). rhGal-9 increased the frequency of these exhausted-like populations, even in the presence of αPD-1, not with the addition of αTim-3. Thus, Gal-9/Tim-3 pathway may promote CD8^+^ exhaustion even in the presence of αPD-1.

## 4 Discussion

Gal-9 role in GC biology has been understudied and there are contradicting studies. Gal-9 expression was associated with improved survival, but only when late stages were ruled out (36). Similarly, cytoplasmic Gal-9 in GC cell lines suppresses migration, invasion, and epithelial-mesenchymal transition and inhibits metastasis (37). Controversially, we observed that high levels of LGALS9 significantly associate with poor outcomes and that extracellular Gal-9 effectively induces cell migration and invasion through its sugar binding domains. Similarly, other authors found that Gal-9 associates with poor prognosis on an Asian cohort of patients (31). This increase in invasion was not only observed on cancer cells but also on gastric epithelial cells, suggesting that this could be in fact a physiological function of extracellular Gal-9. Taken together, it is likely that Gal-9 has different functions depending on its subcellular expression, where cytoplasmic and extracellular Gal-9 display different biological functions.

CD8^+^ T cells are key elements of the cancer immune response, and their dysfunctional states are widely associated with poor clinical outcomes and immunotherapy resistance (38). A reduced effector function, including the production of TNF, IL-2 and IFNγ has been previously reported in dysfunctional CD8^+^ T cells (39–42). It must be noted that dysfunctional T cells are a highly heterogeneous population. Taking this into consideration, it has been suggested that this T cell subset can be classified into ‘pre- dysfunctional’, ‘early dysfunctional’ and ‘late dysfunctional’ cells (43). However, it must be kept in mind that T cell dysfunction is not a binary state, but rather a continuum of different states that culminate into a terminally exhausted phenotype leading to senescence. The transcriptomic analysis brought to light that a molecular signature of dysfunctional CD8^+^ positively correlates with tumor LGALS9, supporting that Gal-9 is associated with CD8^+^ dysfunction in GC. To further evaluate this hypothesis, we performed *in vitro* experiments with T cell primary cultures. Gal-9 increased both PD-1^+^Tim-3^+^ and PD-1^+^Tim-3^+^Lag3^+^ populations, which have been respectively suggested as early dysfunctional and late dysfunctional T cells. Importantly, the appearance of these sub-populations was accompanied by a dose-dependent impairment of cell division. It has been proposed that CD8^+^ T cells retain proliferative capacity during their transition from the pre-dysfunctional state to an early dysfunctional state but lose this capacity at the stage of more profound, ‘late’, dysfunction, either because of an intrinsic block or because their high inhibitory receptor expression suppresses T cell activation (40, 43–47). Herein, we demonstrate that CD8^+^ T cell proliferative capacity is impaired by Gal-9, and that it further drives an exhausted-like phenotype, increasing the frequency of PD-1^+^Tim-3^+^ exhausted-like progenitors and terminally exhausted cells characterized by the expression of Lag3 and failed IFNγ production. Moreover, co-treatment with a Tim3- blocking antibody prevented this effect. Therefore, it is likely that Gal-9, acting through Tim-3 engagement, is sufficient to increase the frequency of these populations. Validating these findings, previous reports show that blockade Gal-9 or Tim-3 restores CD8^+^ T cell proliferation (48). A variety of transcription factors regulate CD8+ T cell dysfunctionality, including TOX and TCF1 [Reviewed in (43)]. TCF1 and TOX appear to be determinants for T cell fate regarding a commitment into a stem-like phenotype or a dysfunctional phenotype in murine models (49). Since our results indicate that Gal-9 is driving the differentiation process into late- dysfunctional cells, further studies should evaluate if this protein can regulate these transcription factors and furthermore and increase the expression of the late-dysfunction marker CXCL13 (40, 43).

Tumour-resident T_reg_ cells have been shown to counteract tumor-specific immune responses by suppressing the infiltration and anti-tumor activity of CD8+ T cells (50). Moreover, T_reg_ accumulation has been associated with immunotherapy resistance in mice and patients (51, 52). Therefore, we evaluated if LGALS9 levels were also associated with increased T_reg_ infiltration. Strikingly, transcriptomic analysis revealed a strong positive correlation, suggesting that Gal-9 could increase Treg infiltration, possibly by expanding this population or alternatively increasing their trafficking into the tumor. Seki et al. showed that Lgals9 knockout (KO) mice displayed a reduced frequency of T_reg_ (52). A separate study showed that this KO mouse had impaired Foxp3 expression, that exogenous Gal-9 could restore Foxp3 function, and furthermore, that TGFβ1 induces Gal-9 expression in a feedforward loop (53). In addition, Gal-9 promoted TGFβ1-dependent induction of T_reg_ via the TGFβ/Smad signaling pathway. Given the evidence from mice models and our bioinformatics results, as a proof of concept we evaluated if Gal-9 could expand T_reg_, using T cell primary cultures from healthy donors. In accordance with our hypothesis, we observed that Gal-9 at the three concentrations used effectively increased T_reg_ frequency. Furthermore, Gal-9 was able to upregulate Foxp3 expression, enrich the Tim3^+^T_reg_ population and potentiate iT_reg_ suppressive capacity.

It has been shown that Tim-3 blockade relieves T_reg_ mediated immunosuppression. Using the Tgfbr1/Pten double KO mouse model, the application of an αTim- 3 antibody reduced T_reg_ count and restored IFNγ production haltering tumor growth (27, 28). Tim-3 appears not only to play a key role on Treg function in cancer, but also in autoimmune diseases such as osteoarthritis, where a reduction of Tim-3 expression on T_reg_ associates with a decreased production of IL-10 (26). Besides Tim-3, Gal-9 has been reported to act through other receptors such as CD44 and CD137 (54). In murine models where Gal-9 could expand Treg, this action was principally attributed to an interaction with the CD44 receptor that enhanced the stability and function of these cells (53). However, our observations in human peripheral T cells, Gal-9 action appears to be predominantly mediated through the Tim-3 receptor, although we cannot rule out that Gal-9 could also, albeit partially, mediate changes through alternative receptors. Mechanistically, it has been proposed that Tim3 can enhance Treg effector-like phenotype through glycolytic metabolism (PMID: 34525351). In accordance, Gal-9 has been proposed to regulate T cell function through the PI3K/AKT/mTOR pathway (PMID: 38141929). Taking together it is possible that Gal-9 signaling through Tim-3 modulates cell metabolism by fine-tuning of the PI3K/AKT/mTOR pathway. While this remains to be evaluated, we can conclude that Tim-3 is required for Gal-9 mediated T_reg_ expansion.

In the field of immunotherapy, there remains an open question of whether ICB acts by reinvigorating a tumor-resident T cell population or mobilization of T cells from outside the tumor also occurs. Studies in mice models revealed that stem-like T cells were critical for obtaining a positive tumor response upon application of single agent αPD-1 therapy or αPD-1 and αCTLA-4 combination therapy. However, by depleting stem-like T cells and thus leaving late-dysfunctional T cells, treatment with ICB still reduced tumor growth (14), thus indicating that this treatment not only prevents exhaustion but can also rescue exhausted cells. Other studies presented similar results (40, 46), supporting that early-dysfunctional T cells, but not late-dysfunctional T cells, may be responsible for the favorable responses observed with ICB therapies [Extensively reviewed in (43)]. Tim-3 targeting promotes IL-12-dependent antitumor immunity without altering immune infiltration (55), thus supporting a key role for this pathway in the peripheral activation of T cells and possibly early fate establishment during antigen presentation.

**Figure 6:**
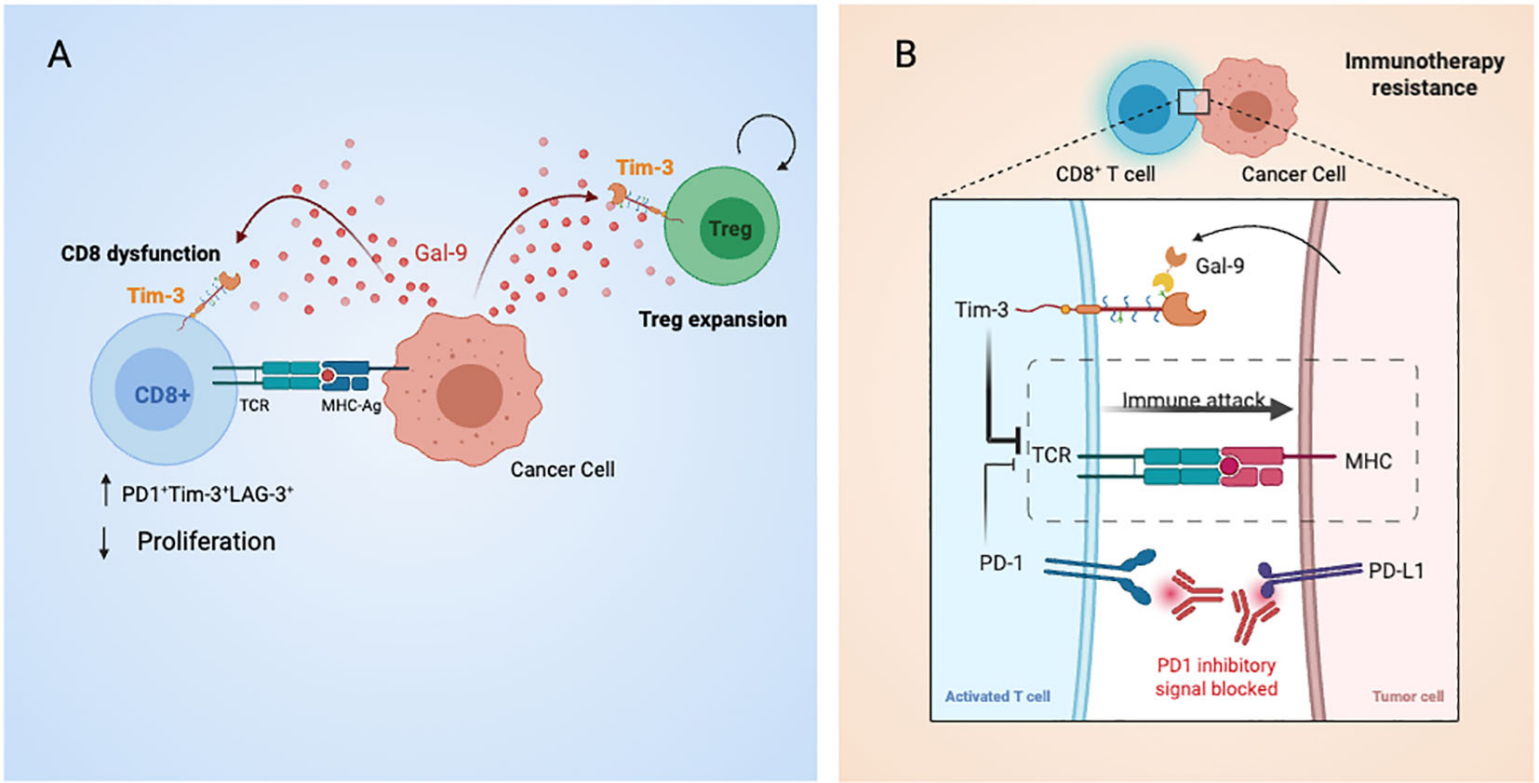
Graphical summary of Galectin-9-mediated immune modulation and checkpoint blockade bypass in gastric cancer. **(A)** Tumor cells secrete Galectin-9, which binds to Tim-3 on CD8^+^ T cells and Tregs, promoting T cell exhaustion and enhancing Treg suppressive activity. These effects contribute to immune evasion and resistance to PD-1 blockade. **(B)** Cell-to-cell interactions between antigen-presenting cells and T cells are shown, including TCR–MHC engagement and co-inhibitory signaling via PD-1 and Tim-3. Dual blockade with anti-PD-1 and anti-Tim-3 antibodies is proposed to restore effector T cell function and counteract Gal-9-driven immunosuppression.

Herein we report that a CD8^+^ dysfunction molecular signature is positively correlated with LGALS9 levels. Importantly, the same signature applied to pre-treatment transcriptomic data from patients with melanoma who subsequently received ICIs, consistently out-performed all other candidate predictive biomarkers tested, including PD-L1 levels, tumor mutational burden, and an IFNγ signature (33). Given the results of our bioinformatic analysis, we evaluated if Gal-9 could provide a bypass to the presence of a PD1 blocking antibody. Even in presence of the αPD-1, Gal-9 increased the frequency of both the early (PD-1^+^Tim3^+^) and late (PD-1^+^Tim3^+^Lag3^+^) exhausted phenotypes. The participation of the Tim-3 receptor is highlighted by the effectively decreased frequency of these subsets. Of note, it was published that Gal-9 could also bind to PD-1, in a way that promoted a lattice formation between PD-1 and Tim-3, which was proposed to increase the stability of dysfunctional T cells, possibly by reducing Tim-3 mediated cell death (56). These results have clinical applicability as they suggest that the axis Gal-9/Tim-3 T cells can push T cells into a dysfunctional state even in the presence of an ICB antibody (**Figure 6**). Interestingly these results indicate not only that Gal-9 presence could provide a bypass to αPD-1 therapy and thus contribute to ICB resistance, but also shine a light on αPD-1’ mechanism. In our experiments, αPD-1 treatment effectively reduced the amount of dysfunctional T cells and thus a principal modus operandi of αPD-1 therapies may be to obstruct the differentiation of T cells into a dysfunctional state. Although the main discussion within the field has been that a durable response to α-PD1 therapy requires the presence of tumor-specific T cells with low levels of dysfunction, our results point to another possibility. Whether it is merely the levels of exhaustion, or also the presence of inhibitory ligands that are the determinants is yet to be ascertained. As T cell exhaustion is led by a robust system of inhibitory pathways, under the activation of other co-inhibitory pathways such as Gal-9/Tim-3, T cells may undergo this differentiation regardless of the αPD-1 treatment. In line with this idea, an immuno-predictive score for neuroblastoma based on the levels of inhibitory and activating immune-checkpoint genes, IMPRES, was found to be associated with immunologically hot tumors and longer overall survival in patients with untreated metastatic melanoma (57). In predicting responses to ICB in this setting, IMPRES strikingly achieved an overall accuracy of AUC = 0.83, outperforming existing predictors and capturing almost all true responders while misclassifying less than half of the non-responders. This work strengthens the hypothesis that a robust network of regulatory checkpoints is in operation to maintain the tumor immunosuppressive microenvironment (**Figure 6**). Noteworthy, our results showed that a combinatory strategy using αPD-1 together with αTim-3 effectively prevented the effects of Gal9 on T cell fate, further supporting the idea of combining ICB for cancer treatment. Encouragingly, there are 63 clinical trials of antibodies targeting Tim-3 either alone or in combination with PD-1 blockers. Moreover, Gal-9 is a secreted protein that can be detected in plasma, and thus future studies should examine the clinical benefit of evaluating Gal-9 and intra-tumor Tim-3 levels as a companion diagnostic for αPD-1 + αTim-3 combinatorial immunotherapy. Furthermore plasmatic Gal-9 may prove value as a resistance risk indicator for patients receiving ICB monotherapy and could signal when to begin combinatorial therapy.

In summary, this study combines transcriptomic analysis with ex vivo functional assays to uncover a role for Galectin-9 in promoting CD8^+^ T cell dysfunction and Treg expansion in gastric cancer. A major strength is the integration of patient-derived data with mechanistic *in vitro* validation. However, reliance on the TCGA database, which lacks treatment metadata and may introduce selection bias, limits the ability to generalize findings to treated populations. Further *in vivo* and clinical studies are warranted to confirm these observations.

## Data Availability

The raw data supporting the conclusions of this article will be made available by the authors, without undue reservation.
