# Supplementary material for: Galectin-9 and Tim-3 in gastric cancer: a checkpoint axis driving T cell exhaustion and Treg-mediated immunosuppression independently of anti-PD-1 blockade
Source: Front Immunol. 2025 Jul 1;16:1600792. doi: 10.3389/fimmu.2025.1600792 (PMC12259562; doi:10.3389/fimmu.2025.1600792)
Supplement: Supplementary file 1 [file DataSheet1.pdf]

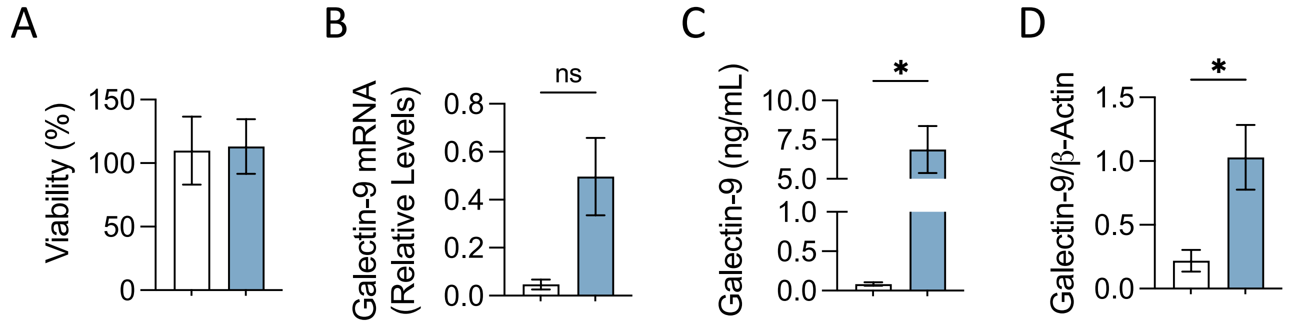

Supplementary Figure 1. Galectin-9 gain of function in AGS cell line increases Gal-9 expression and secretion.
*$p<0.05$, U-Man Whitney, n=3.
