# Supplementary figures and images for: Galectin-9 and Tim-3 in gastric cancer: a checkpoint axis driving T cell exhaustion and Treg-mediated immunosuppression independently of anti-PD-1 blockade
Source: Front Immunol. 2025 Jul 1;16:1600792. doi: 10.3389/fimmu.2025.1600792 (PMC12259562; doi:10.3389/fimmu.2025.1600792)

A

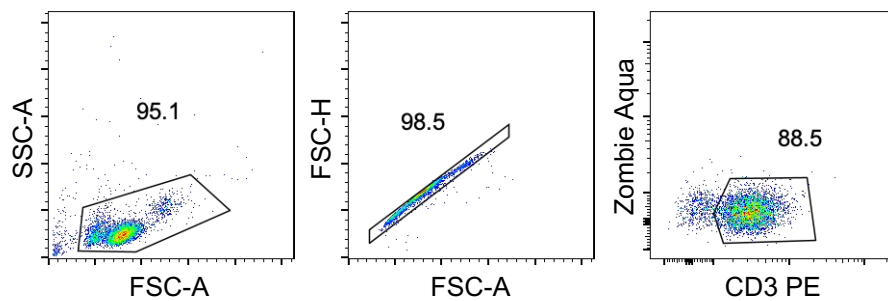

B

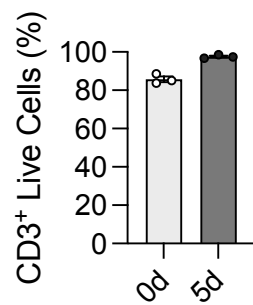

C

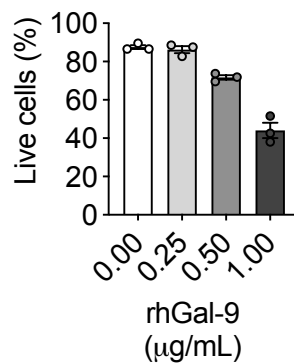

D

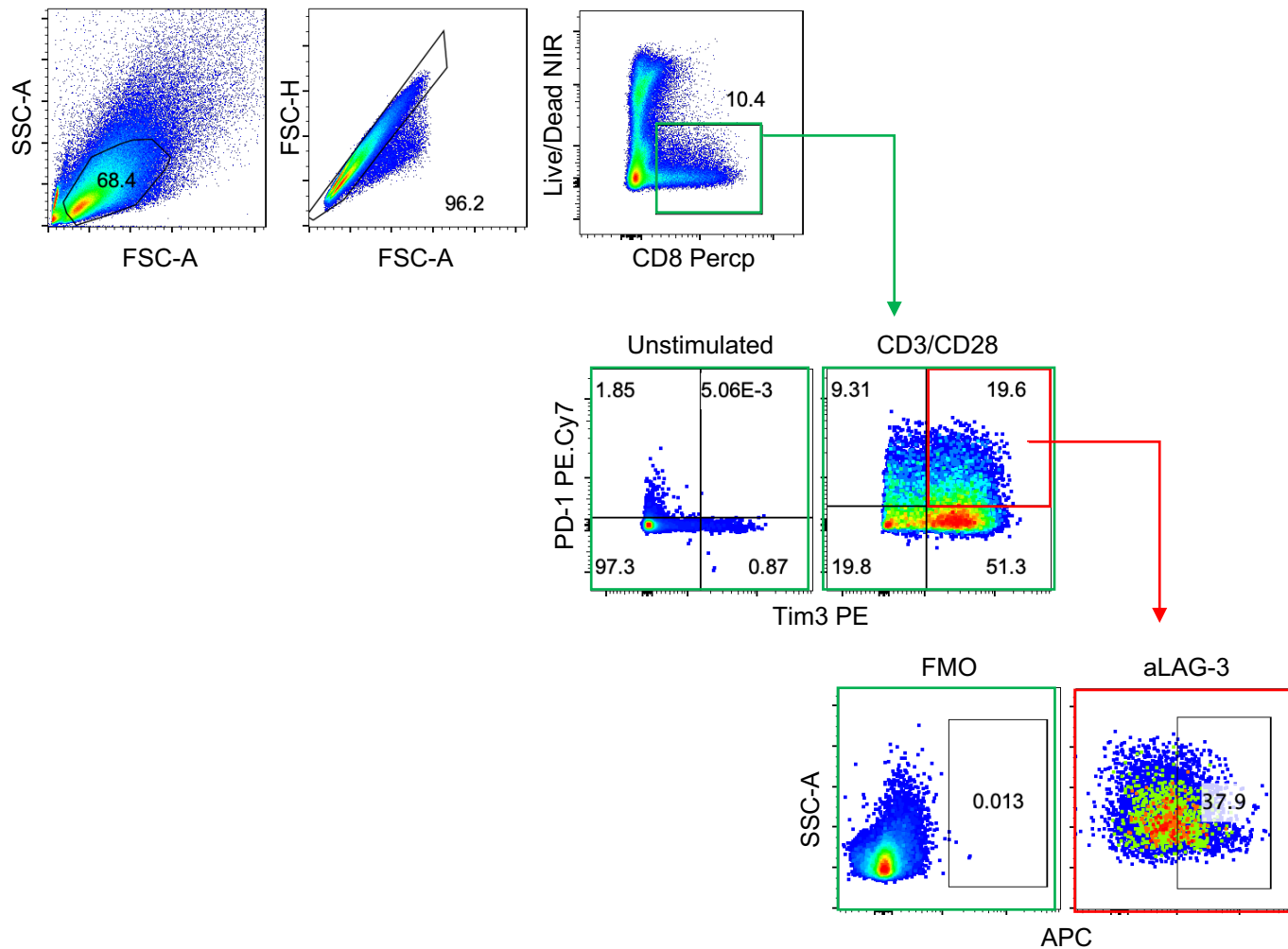

Supplement: Supplementary file 2 [file DataSheet2.pdf]

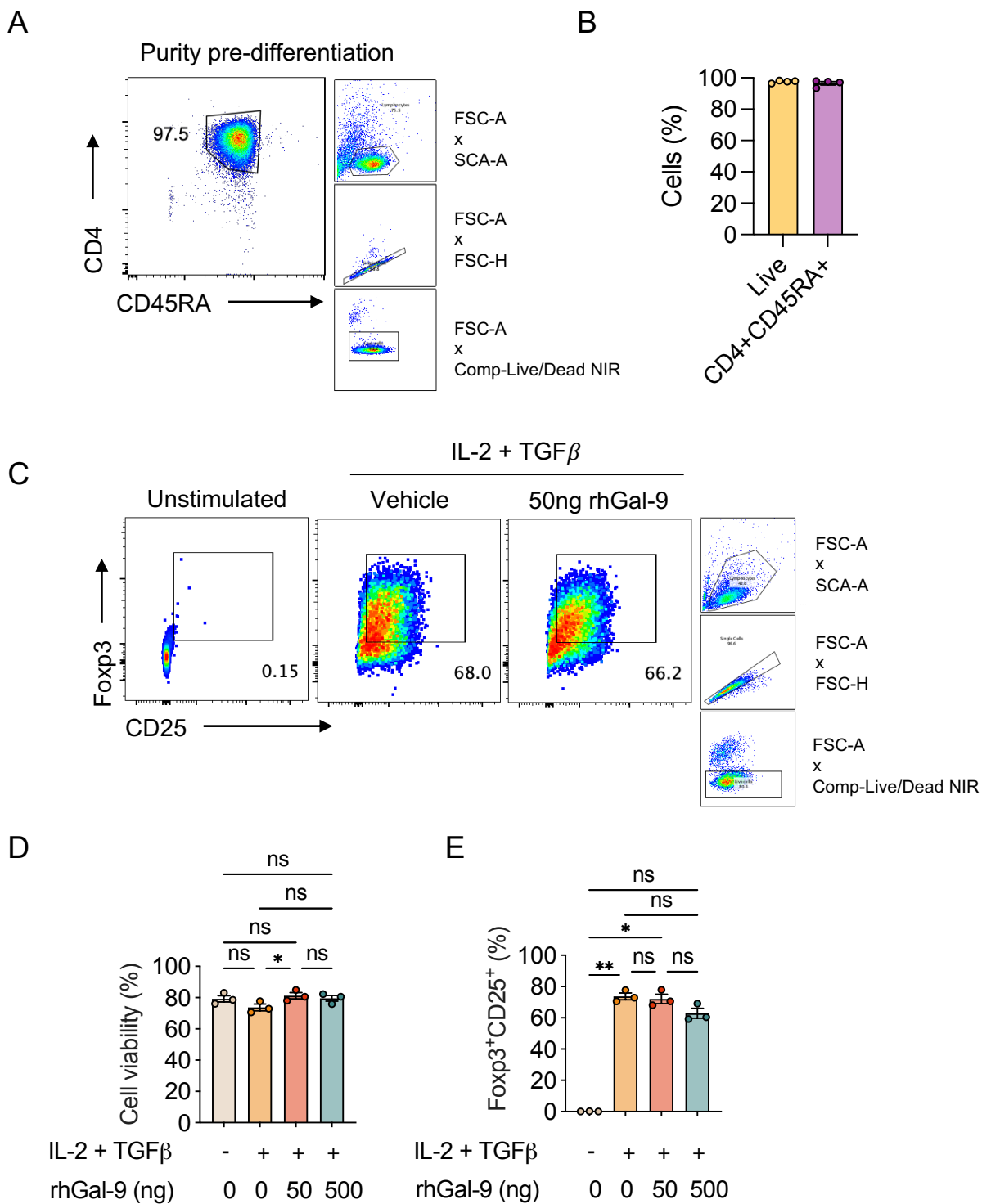

Supplementary Figure 3. Isolation of Naïve CD4<sup>+</sup> T cells and Treg differentiation in vitro.

Supplement: Supplementary file 3 [file DataSheet3.pdf]
