# Supplementary material for: Galectin-9 and Tim-3 in gastric cancer: a checkpoint axis driving T cell exhaustion and Treg-mediated immunosuppression independently of anti-PD-1 blockade
Source: Front Immunol. 2025 Jul 1;16:1600792. doi: 10.3389/fimmu.2025.1600792 (PMC12259562; doi:10.3389/fimmu.2025.1600792)
Supplement: Supplementary file 4 [file DataSheet4.pdf]

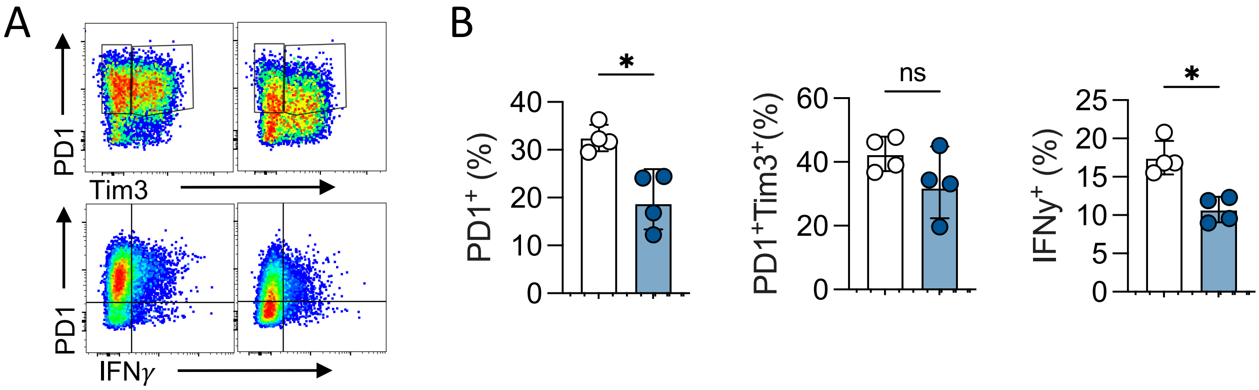

Supplementary Figure 4. Galectin-9 reduces IFNγ production and PD1^{+} single positive CD8+ T cell frequency. CD8^{+} T cells were isolated from healthy donors PBMCs by magnetic enrichment and activated with aCD3/CD28 beads in the presence of 50ng/mL rhGal-9. After 96h, cells were harvested and stained for flow cytometry to analyze PD1, Tim3 and IFNγ expression on CD8^{+} live cells. Flow cytometry analysis on CD8^{+} Live cells is shown (A,B). n=4, *$p<0.05$, Mann-Whitney test.
